# Antiproliferative Activity of Neem Leaf Extracts Obtained by a Sequential Pressurized Liquid Extraction

**DOI:** 10.3390/ph11030076

**Published:** 2018-07-30

**Authors:** Klebson S. Santos, Andriele M. Barbosa, Victor Freitas, Ana Veruska C. S. Muniz, Marcelo C. Mendonça, Ricardo C. Calhelha, Isabel C. F. R. Ferreira, Elton Franceschi, Francine F. Padilha, Maria Beatriz P. P. Oliveira, Cláudio Dariva

**Affiliations:** 1 NUESC/ITP, Program in Industrial Biotechnology-Tiradentes University, Aracaju 49032-490, Brazil; andrielemendonca@yahoo.com.br (A.M.B.); marcelo_costa@unit.br (M.C.M.); franceschi.elton@gmail.com (E.F.); fpadilha@yahoo.com (F.F.P.); claudio.dariva@gmail.com (C.D.); 2 REQUIMTE/LAQV, Department of Chemistry Sciences, Faculty of Pharmacy, University of Porto, 4050-313 Porto, Portugal; beatoliv@ff.up.pt; 3 Chemistry Investigation Centre (CIQ), Department of Chemistry, Faculty of Sciences, University of Porto, 4169-007 Porto, Portugal; vfreitas@fc.up.pt; 4 Embrapa Coastal Tablelands, Aracaju 49025-040, Brazil; ana.veruska@embrapa.br; 5 Mountain Research Center (CIMO), School of Agriculture, Polytechnic Institute of Bragança, Campus de Santa Apolónia, 5300-253 Bragança, Portugal; calhelha@ipb.pt (R.C.C.); iferreira@ipb.pt (I.C.F.R.F.)

**Keywords:** sequential pressurized liquid extraction, neem leaves, antiproliferative activity

## Abstract

*Azadirachta indica* A. Juss (neem) extracts have been used in pharmaceutical applications as antitumor agents, due to their terpenes and phenolic compounds. To obtain extracts from neem leaves with potential antiproliferative effect, a sequential process of pressurized liquid extraction was carried out in a fixed bed extractor at 25 °C and 100 bar, using hexane (SH), ethyl acetate (SEA), and ethanol (SE) as solvents. Extractions using only ethanol (EE) was also conducted to compare the characteristics of the fractionated extracts. The results obtained by liquid chromatography-electrospray ionization mass spectrometry suggested a higher concentration of terpenes in the SEA extract in comparison to SH, SE, and EE extracts. Therefore, antiproliferative activity showed that SEA extracts were the most efficient inhibitor to human tumor cells MCF-7, NCI-H460, HeLa, and HepG2. Hepatocellular cells were more resistant to SH, SEA, SE, and EE compared to breast, lung, hepatocellular, and cervical malignant cells. Neem fractioned extracts obtained in the present study seem to be more selective for malignant cells compared to the non-tumor cells.

## 1. Introduction

Neem (*Azadirachta indica* A. Juss) is a tree of the Meliaceae family found worldwide in semi-tropical and tropical climates [1]. The medicinal properties of the plant are related to the presence of salannin, nimbin, gedunin and nimbolide [2], among others terpenes and phenolic compounds in neem leaves extracts [3,4]. Neem-compounds have exhibited chemopreventive and anticancer efficacy due to their cellular and molecular mechanisms of action, such as immunomodulatory, carcinogen-detoxification, cell-cycle arrest, programmed cell death, and anti-metastatic [5]. The anticancer activity of neem constituents can inhibit the growth of a variety of human cancers, such as lung, breast, oral, prostate, skin, liver [6,7] and cervical [8].

Pharmacological bioactive compounds can be obtained by different extraction methods such as maceration, soxhlet, and pressurized liquid extraction (PLE) [9,10]. PLE shows a great potential for the extraction of metabolites from vegetable matrices [11,12,13] due to the possibility of using a variety of polar and non-polar solvents under high pressure, which improves the efficiency of the extraction process [14,15]. PLE enables extraction in a lower extraction time and using a small amount of solvent [16,17], and was also used for the exhaustive extraction of analytes in one or more clean-up steps [18]. In this sense, the PLE is considered a promising process to obtain natural compounds [11,12,13].

As the majority of vegetable extracts, the neem extract also is composed by a variety of chemical compounds. In this sense, depending on the characteristics of the extraction solvent used, the potential medicinal activity of the extract can also be quite distinct [4,10]. Hexane, ethyl acetate, and ethanol are efficient solvents used to extract terpenes and flavonoids, that are important compounds to human health [19,20]. The aim of this study was to develop a method to obtain extracts with antiproliferative effects from neem leaves, by using a sequential process of pressurized liquid extraction employing hexane (SH), ethyl acetate (SEA), and ethanol (EE) as solvents, and evaluate the cytotoxicity of the extracts obtained against human tumor cell lines and non-tumor liver cells.

## 2. Results and Discussion

### 2.1. Pressurized Liquid Extraction Process

Table 1 presents the average values and standard deviation of the extraction yield of neem leaves using n-hexane (SH), ethyl acetate (SEA), and ethanol (SE) as solvents in the sequential pressurized liquid extraction and the ethanolic extract solvent (EE) using the one-step pressurized liquid extraction. The results presented in Table 1 showed that increasing the solvent polarity from hexane to ethanol (80%) leads to a significant enhancement in the dry mass (yield) obtained from neem leaves. Furthermore, SE and EE yields were not significantly different at *p* < 0.05, suggesting that the previous extractions with hexane and ethyl acetate did not reduce the ethanol extractive capacity. However, either hexane and ethyl acetate showed a lower capacity to obtain a dry extract from neem leaves compared to ethanol.

### 2.2. Liquid Chromatography Analysis

**Figure 1:**
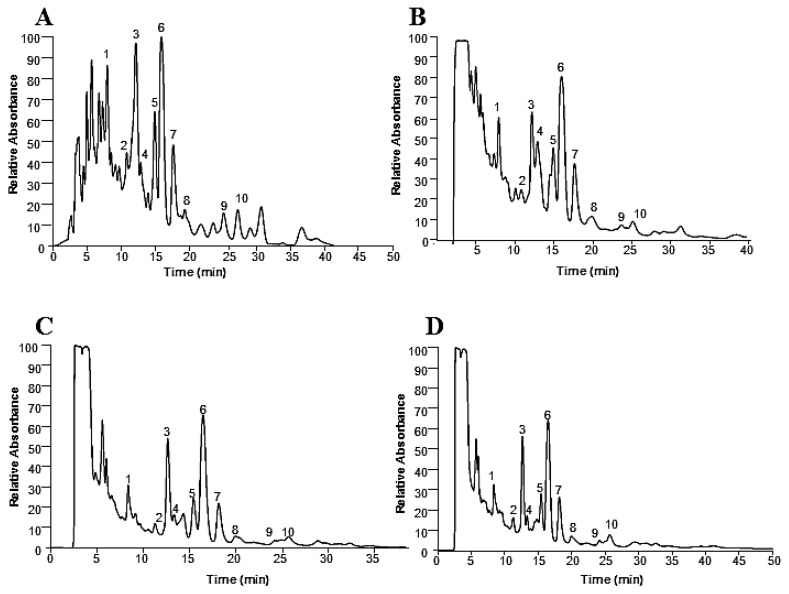
LC-PDA chromatograms at 210–220 nm of the neem leave extracts obtained by PLE. SH (**A**), SEA (**B**), SE (**C**), and EE (**D**).

In this study, liquid chromatography-mass spectrometry was used for the chemical characterization of the neem leaves extracts. Figure 1 shows the PDA chromatograms of neem extracts obtained by PLE with different solvents. 

Table 2 presents the 10 neem leaves compounds tentatively identified by ESI-MS from their fragmentation (*m*/*z*), in positive mode, with the respective HPLC areas for the different extraction solvents.

**Figure 2:**
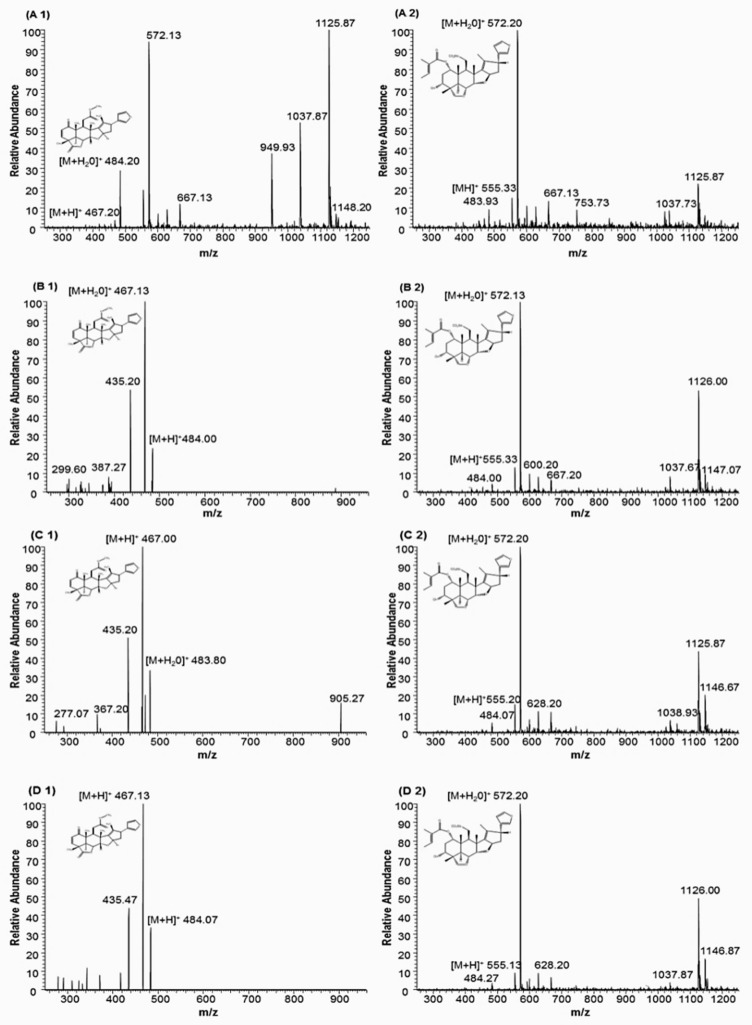
Mass spectra of nimbolide (**A1**, **B1**, **C1**, and **D1**) and 3-Deacetylsalannin (**A2**, **B2**, **C2**, and **D2**) terpenoids extracted by pressurized liquid extraction. Capital letters A, B, C, and D correspond to SH, SEA, SE, and EE, respectively.

It can be observed from Figure 1 and Table 2 that the compounds obtained in each extraction fraction were similar. On the other hand, the concentration of each compound was distinct related to the polarity and capacity of solvation of each solvent. Nevertheless, it should be noted that different compounds in different extracts may result in distinct antiproliferative activity. Also, distinct concentrations of biocompounds in the extracts can alter their antiproliferative potential. The compounds extracted by SH and SEA were similar, but with different relative absorbance, as could be observed in Figure 1 and Table 2. The extracts from SH and SEA are more concentrated in the compounds in comparison to SE and EE extracts. Peak 6 is the most abundant and its mass spectral analysis suggested that it corresponds to nimbolide or 3-deacetylsalannin. Figure 2 presents the mass spectra and the respective structure of these compounds.

These compounds have already been described in neem leaves [5,21,22,23,24,25,26,27,28,29,30]. Figure 1A,B show a relatively higher absorbance (about 20%) for the peaks 1, 3 and 6. Accordingly, these solvents (hexane and ethyl acetate) have a low contribution to overall extraction yield, but a high contribution for several compounds (this is the case of 1, 3 and 6). Comparing the solvents, it seems that the chemical profile of the polar solvents presents more similarity among them when compared with the non-polar one (Figure 1A). 

The mass of nimbolide is 466.199 with a molecular formula C_27_H_30_O_7_, and the standard shows a mass spectrum [M + H]^+^ peak at *m*/*z* 467.211 [21]. In Figure 2, the spectral analysis shows a [M + H]^+^ peak corresponding to nimbolide in all obtained extracts. The molecular formula C_32_H_42_O_8_, corresponding to 3-deacetylsalannin [M + H]^+^, has been identified in neem leaves [22]. The neem compounds identified by LC-MS show the ability to make adducts with H_2_O, forming an additional fragment [M + 18]^+^. Other fragments can result from the rupture of ester bonds from the [M + H]^+^ species [31], thereby corroborating the identification of some compounds from Table 2.

Among the 10 compounds identified in Table 1, just the compound 4 (peak 4) was not a terpene: it corresponds to rutin, a flavone [M + H]^+^ at m/z 611 [32]. However, the terpenes obtained in this study are more soluble in less-polar solvents such as n-hexane and ethyl acetate compared with the polar solvent ethanol. The affinity of the targeted compounds with the solvent used in the extraction is very important to obtain bioactive compounds such as anthocyanins, flavones, and terpenes [9,33]. According to the results, ethyl acetate (SEA) and n-hexane (SH) seem to be good options to obtain terpenes from neem leaves by sequential pressurized liquid extraction. Moreover, in this study, it was also demonstrated that the sequential extraction in fixed bed extractor cell using SEA in the second step improves the extraction of terpenes such as nimbolide and 3-deacetylsalannin, compared to the other solvents.

### 2.3. Cytotoxicity Evaluation of Neem Leaves Extracts 

Neem extracts have demonstrated activity against tumor cells [14,34]. Due to the lack of studies analyzing the influence of distinct fractions of neem extracts in human tumor cells, it was investigated the antiproliferative profile of neem extracts fractions against four human tumor cells and one non-tumor cell lines. The results obtained are summarized in Table 3.

As presented in Table 3, all neem extracts could inhibit the growth of human tumor cell lines. Nevertheless, these extracts exhibit different values of GI_50_. SEA extracts show the highest potential to inhibit the growth of tumor cells, presenting GI_50_ value smaller than those found for SH, SE, and EE. The result suggests that the clean-up process performed by the sequential PLE extraction was able to produce fractions with high antitumor effects. NCI-H460, HeLa, and HepG2 cells were more sensitive to SEA than the other studied cells. Some investigations have demonstrated that plant-derived fractions obtained by high pressure show an antiproliferative potential against cancer cells [35,36,37,38,39]. The results obtained in this study are in agreement with Hao et al., who reported that neem extracts have a potential therapeutic effect on the growth of various types of cancer cells [34].

Sharma et al., tested a vast range of concentrations (10–500 µg/mL) of ethanolic neem leaf extract against MCF-7 and Hela, and their GI_50_ values were of 350 µg/mL on MCF-7 cells and 175 µg/mL on HeLa cells. Nevertheless, these GI_50_ values were higher than GI_50_ values found in the present study for SEA, that were 82.3 ± 4.3 µg/mL and 48.8 ± 4.3 µg/mL on MCF-7 and Hela, respectively. Moreover, SEA extract concentrations were also more cytotoxic to the MCF-7 and HeLa cells than 50 and 100 µg/mL of neem ethanolic extract combined with 5 µM cisplatin (antitumor agent). According to Sharma et al., these combinations have a synergistic effect on cancer cell growth inhibition in 52.2 (MCF-7) and 65% (HeLa) [8]. This higher antiproliferative activity exhibited by the SEA extract can be suggested due to the higher selectivity of the ethyl acetate solvent to obtain cytotoxic compounds from neem leaves. Furthermore, the 10 biocompounds obtained by the sequential process of pressurized liquid extraction may be acting in synergistic effect among them, contributing for the higher SEA extract cytotoxicity.

SEA (Table 3) also exhibits a higher cytotoxic effect against human tumor cells compared to the methanolic extracts reported by Pereira et al., who obtained GI_50_ values with 83 ± 9 (MCF-7), 262 ± 4 (NCI-H460), 160 ± 13 (HeLa) and 100 ± 10 (HepG2) µg/mL of *Thymus vulgaris* leaves and 154 ± 7 (MCF-7), 229 ± 16 (NCI-H460), 224 ± 12 (HeLa) and 111 ± 12 (HepG2) µg/mL of *Mentha x piperita* leaves [40]. According to Hao et al., the antiproliferative activity of neem extract has been associated with the suppression of angiogenesis, induction of cell death, and enhancement of immune response against malignant cells [34].

Non-tumor liver PLP2 cells have been used to evaluate the tumor selectivity effect [40,41]. As can be observed in Table 3, these non-tumor cells were more resistant than the human tumor cells to the treatment with SH, and SEA. This result can contribute to the alternative therapy development against the growth of malignant cells. Our results corroborating the Donno and co-authors finds that suggested that the phenolic and terpenic compounds from plants are biologically active substances [42].

## 3. Materials and Methods 

### 3.1. Neem Samples

Young and old leaves of neem (*Azadirachta indica* A. Juss) were collected during the spring from Germplasm Bank (GBN) of Embrapa Coastal Tablelands (Sergipe, Brazil). All leaves were mixed and dried at 45 °C for 36 h in an oven with hot-air circulation. After that, the leaves were milled and classified according to its size in the range of 8 to 16 mesh using the Tyler sieves series. The material was stored under refrigeration and protected from light until the extractions. 

### 3.2. Pressurized Liquid Extraction Process

The PLE runs were performed in a 100 mL high-pressure extractor cell using 20 g of neem leaves. The extractor was constructed in stainless steel and was coupled to two high pressure pumps to the continuous displacement of high-pressure solvents. The extractor cell has a jacket and it was connected to a recirculating ultrathermostatic bath to temperature control. Pressure transducer and universal process indicators were used to monitor the extraction process variables. After inserting the neem leaves in the extractor, it was connected to the experimental unit and the first PLE step was performed at 100 bar and 25 °C, using n-hexane as a solvent during 60 min at a flow rate of 1 mL/min. After completed this step, the flow of hexane was interrupted in the high-pressure pump, and the system was continuously flushed with carbon dioxide (around 30 bars) during 10 min to remove residual n-hexane. After that, the system was depressurized and the second PLE step was performed at 100 bar and 25 °C, using ethyl acetate as an extraction solvent for 60 min at 1 mL/min. The system was then flushed again with carbon dioxide during 10 min to remove residual ethyl acetate. The third PLE step was conducted using water/ethanol (20:80 v/v) mixtures as solvent at 100 bar, 25 °C, 1 mL/min for 60 min of extraction. One-step pressurized liquid extraction using water/ethanol (20:80 v/v) mixtures as a solvent was also carried out for analyzing the effects of the sequential extraction sequential. After removing the organic solvents of the obtained solution in a rota-evaporator, the neem extracts were named SH, SEA, and SE for the sequential process using n-hexane, ethyl acetate, and water/ethanol as solvent, respectively. The one-step PLE produced an extract named EE. All extractions were performed in triplicate.

### 3.3. HPLC-PDA-ESI-MS Analysis

Liquid chromatography-mass spectrometry has been used to characterize metabolic in the pharmaceutical analysis [21]. In this study, the extracts were analyzed by HPLC-PDA-ESI-MS using a Finnigan Surveyor Plus (Finnigan Corp. San José, CA, USA) High-Performance Liquid Chromatography (HPLC) system fitted with a photodiode array (PDA, at 210–220 nm) and a liquid chromatography quaternary pump. The system was coupled to a Finnigan LCQ Deca XP max mass detector equipped with electrospray ionization source (ESI). A LIChroCART^®^ RP-18 column (150 mm × 4.6 mm, 5 µm) (Merck Millipore, Darmstadt, Germany) was used. The mobile phase was acetonitrile/water (60:40 *v*/*v*) at a flow rate of 0.50 mL min^−1^, and the runtime was 40 min with a sample volume injection of 25 µL. The mass spectrometry analysis was performed under positive electrospray ionization (ESI+). The mass spectra were obtained in the scan range of 250–1200 *m*/*z* [21], controlled by Xcalibur software version 2.2.

### 3.4. Cytotoxicity Assays 

The cell lines used were: MCF-7 (breast adenocarcinoma), NCI-H460 (non-small cell lung cancer), HeLa (cervical carcinoma), HepG2 (hepatocellular carcinoma) and PLP2 (non-tumor liver primary culture). Each cell line was grown in a 96-well microplate, at a density of 7.5 × 10^3^ cells/well for MCF-7 and NCI-H460, and 1.0 × 10^4^ cells/well for HeLa, HepG2, and PLP2. The cells were allowed to attach for 24 h. After this period, distinct neem extract concentrations (1.56–250 µg/mL) or ellipticine (positive control) were added to the cells and incubated for 48 h. After that, prechilled trichloroacetic acid (TCA 10%, 100 μL) was added and incubated for 60 min at 4 °C to improve the adherence of the cells. The plates were washed with deionized water, dried and after the addition of a sulforhodamine B solution (SRB 0.1% in 1% acetic acid, 100 μL), the mixture was incubated for 30 min at room temperature. Subsequently, the plates were washed with acetic acid (1%) to remove the unbound SRB and dried. The bounded SRB was solubilized with Tris (10 mM, 200 μL) and the absorbance measured at 540 nm using an ELX800 microplate reader (Bio-Tek Instruments, Inc.; Winooski, VT, USA) [40,43].

### 3.5. Statistical Analysis 

Statistical analysis of the results was conducted by using one-way ANOVA, followed by a post hoc Tukey’s test using Prism version 5.0 software (San Diego, CA, USA). Statistical significance was concluded with *p* < 0.05.

## 4. Conclusions

This study demonstrated that sequential-PLE is an efficient methodology for extraction of bioactive compounds from neem leaves. The use of three different solvents for the extraction process provides extracts with different antiproliferative potential. SEA extract was the most efficient growth inhibitor for the tumor cells, with a GI_50_ dose of 52.3 ± 4.8, 48.8 ± 4.3, 60.6 ± 4.3, and 82.3 ± 4.3 µg/mL to HepG2, HeLa, NCI-H460, and MCF-7, respectively. Nevertheless, results indicated that PLP2 non-tumor cells were more resistant to all extracts obtained in the present study with a GI_50_ dose higher than 200 µg/mL. The present study provides a process to obtain extracts of neem leaves with potential application as antiproliferative of malignant cells.

## Figures and Tables

**Table 1:** Effect of different solvents, hexane (SH), ethyl acetate (SEA), and ethanol (SE and EE) on the dry mass of neem leaves in the PLE process.

Neem Leaves (20 g)	One-Step Extraction (g)	Three-Step Extraction (g)
Hexane (SH)	____	0.07 ± 0.01 ^b^
Ethyl acetate (SEA)	____	0.06 ± 0.01 ^b^
Etanol 80% (SE)	____	1.50 ± 0.12 ^a^
Etanol 80% (EE)	1.58 ± 0.26 ^a^	_____

Data are reported as mean ± standard deviation values. Equal letters (a, and b) indicate that there is no difference between the extractions. Not performed (___).

**Table 2:** Neem leaves compounds in the PLE extracts tentatively identified by ESI-MS from their fragmentation (*m*/*z*), in positive mode, and HPLC areas for the distinct extraction solvents.

Extract	Peak	t_R_ (min)	Area	Compound	Observed Ions (*m*/*z*)
SH	1	8.42	45724118	Nimbandiol	371, 401, 421, 425, 441, 444, 457 [M + H]^+^, 474 [M + H_2_O]^+^
SEA		8.46	38701542		
SE		8.56	20010994		
EE		8.40	25006988		
SH	2	11.29	29873977	6-Deacetylnimbin	389, 453, 467, 499 [MH]^+^, 516 [M + H_2_O]^+^
SEA		11.42	22241890		
SE		11.32	2818278		
EE		11.46	14511268		
SH	3	12.77	81159631	2,3-Dihydronimbolide	178, 315, 426, 433, 441, 450, 469 [MH]^+^, 486 [M + H_2_O]^+^
SEA		12.75	37973099		
SE		12.76	28340365		
EE		12.84	36750310		
SH	4	13.91	13066767	Rutin	266, 480, 546, 558, 611 [M + H]^+^, 628 [M + H_2_O]^+^
SEA		14.02	17659833		
SE		13.95	14990955		
EE		14.02	15674396		
SH	5	15.56	36960991	Nimonol	274, 293, 353, 421, 439, 453 [M + H]^+^, 470 [M + H_2_O]^+^
SEA		15.59	31070403		
SE		15.67	15495619		
EE		15.52	20995715		
SH	6	16.39	70699349	Nimbolide	277, 435, 435, 467 [M + H]^+^,484 [M + H_2_O]^+^
SEA		16.54	86571238		
SE		16.42	50917437		
EE		16.45	57586856		
SH	6	16.39	70699349	3-Deacetylsalannin	555 [M + H]^+^, 572 [M + H_2_O]^+^
SEA		16.54	86571238		
SE		16.42	50917437		
EE		16.45	57586856		
SH	7	18.22	32928497	6-Deacetylnimbinene	363, 393, 409, 441 [M + H]^+^, 458 [M + H_2_O]^+^
SEA		18.18	37398457		
SE		18.12	15714996		
EE		18.32	21712675		
SH	8	19.88	15628192	Nimbanal	221, 265, 339, 345, 405, 428, 451,453, 455, 471, 482,493, 511 [M + H]^+^, 528 [M + H_2_O]^+^
SEA		19.93	23156736		
SE		19.87	6245251		
EE		19.74	11010022		
SH	9	24.96	14175318	Salannin	199, 230, 278, 319, 378, 481, 515, 571, 597 [M + H]^+^, 614 [M + H_2_O]^+^
SEA		24.86	12952957		
SE		24.87	5526287		
EE		24.93	2517812		
SH	10	25.49	17132995	Gedunin	184, 259, 287, 344, 372, 405, 425, 451, 483 [M + H]^+^, 500 [M + H_2_O]^+^
SEA		25.67	13462271		
SE		25.75	6429235		
EE		25.70	7359673		

**Table 3:** Cytotoxicity of neem leaves extracts obtained by PLE against several human cancer cells (MCF-7, NCI-H460, HeLa, and HepG2) and the non-tumor cell (PLP2). All data <250 µg/mL are reported as a mean ± standard deviation, from growth inhibition at 50% (GI_50_).

Lines	Extract (µg/mL)				Control (µg/mL)
	SH	SEA	SE	EE	Ellipticine
MCF-7	188.8 ± 6.4 ^a^	82.3 ± 4.3 ^b^	>250 ^c^	>250 ^c^	0.9 ± 0.1 ^d^
NCI-H460	224.4 ± 14.4 ^a^	60.6 ± 4.3 ^b^	>250 ^c^	>250 ^c^	1.0 ± 0.1 ^d^
HeLa	203.9 ± 13.6 ^a^	48.8 ± 4.3 ^b^	>250 ^c^	>250 ^c^	1.9 ± 0.1 ^d^
HepG2	115.5 ± 14.4 ^a^	52.3 ± 4.8 ^b^	>250 ^c^	>250 ^c^	1.1 ± 0.2 ^d^
PLP2	>250 ^a^	201.3 ± 17.0 ^b^	>250 a	>250 ^a^	3.2 ± 0.7 ^c^

Ellipticine positive control. Equal letters (a, b, c, and d) in the same line indicate that there is no significant difference in the cytotoxic effects (*p* < 0.05).
